# The temperate marine Peruvian Province: How history accounts for its unusual biota

**DOI:** 10.1002/ece3.70048

**Published:** 2024-07-21

**Authors:** Geerat J. Vermeij, Thomas J. DeVries, Miguel Griffin, Sven N. Nielsen, Diana Ochoa, Marcelo M. Rivadeneira, Rodolfo Salas‐Gismondi, Fernanda Valdovinos

**Affiliations:** ^1^ Department of Earth and Planetary Sciences University of California, Davis Davis California USA; ^2^ Burke Museum of Natural History and Culture University of Washington Seattle Washington USA; ^3^ División Paleozoología Invertebrados Museo de La Plata La Plata Argentina; ^4^ Instituto de Ciencias de la Tierra Universidad Austral de Chile Valdivia Chile; ^5^ Centro de Investigación Para el Desarrollo Integral y Sostenible (CIDIS) Universidad Peruana Cayetano Heredio Lima Peru; ^6^ Centro de Estudios Avanzados en Zonas Áridas Coquimbo Chile; ^7^ Departamento de Biologia Marina, Facultad de Ciencias del Mar Universidad Catolica del Norte Antofagasta Chile; ^8^ Departamento de Paleontología de Vertebrados Museo de Historia Natural‐Universidad Nacional Mayor San Marcos Lima Peru; ^9^ Department of Environmental Science and Policy University of California, Davis Davis California USA

**Keywords:** colonization, extinction, latitudinal gradients, Miocene, Pliocene, refuges, South America

## Abstract

The Peruvian Province, from 6° S in Peru to 42° S in Chile, is a highly productive coastal marine region whose biology and fossil record have long been studied separately but never integrated. To understand how past events and conditions affected today's species composition and interactions, we examined the role of extinction, colonization, geologic changes to explain previously unrecognized peculiar features of the biota and to compare the Peruvian Province's history to that of other climatically similar temperate coasts. We synthesized all available data on the benthic (or benthically feeding) biota, with emphasis on fossilizable taxa, for the interval from the Miocene (23–5.4 Ma) and Pliocene (5.4–2.5 Ma) to the present. We outline the history of ecological guilds including primary producers, herbivores, predators, and suspension‐feeders and document patterns of extinction, colonization, and geographic restriction. We identify twelve unusual attributes of the biota, most of which are the result of repeated episodes of extinction. Several guilds present during the Miocene and Pliocene are not represented in the province today, while groups such as kelps and perhaps intertidal predatory sea stars are relative newcomers. Guilds on soft bottoms and in sheltered habitats were severely affected by extinction, whereas those on hard bottoms were most affected by colonists and held their own in diversity. The Peruvian Province has not served as a biogeographic refuge, in contrast to the coasts of Australasia and Argentina, where lineages no longer present in the Peruvian Province survive. The loss of sheltered habitats since the Pliocene explains many of the present‐day peculiarities of the biota. The history of the province's biota explains its unique attributes. High productivity, a rich Southern Hemisphere heritage, and colonization from the north account for the present‐day composition and unusual characteristics of the biota.

## Graphical abstract

Among extant temperate marine biotas, the Peruvian Province is highly unusual in, among other traits, a very high incidence of limpets, gastropods with a labral tooth (to assist in predation), a reversed latitudinal diversity gradient, and the absence of several ecological guilds. These and other peculiarities arise from a history of high‐magnitude extinction during the last fifteen million years. The province is also unusual in not functioning as a biogeographic refuge for previously more widely distributed species.

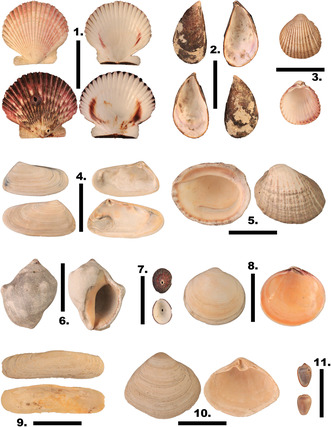

## 1 INTRODUCTION

**FIGURE 1:**
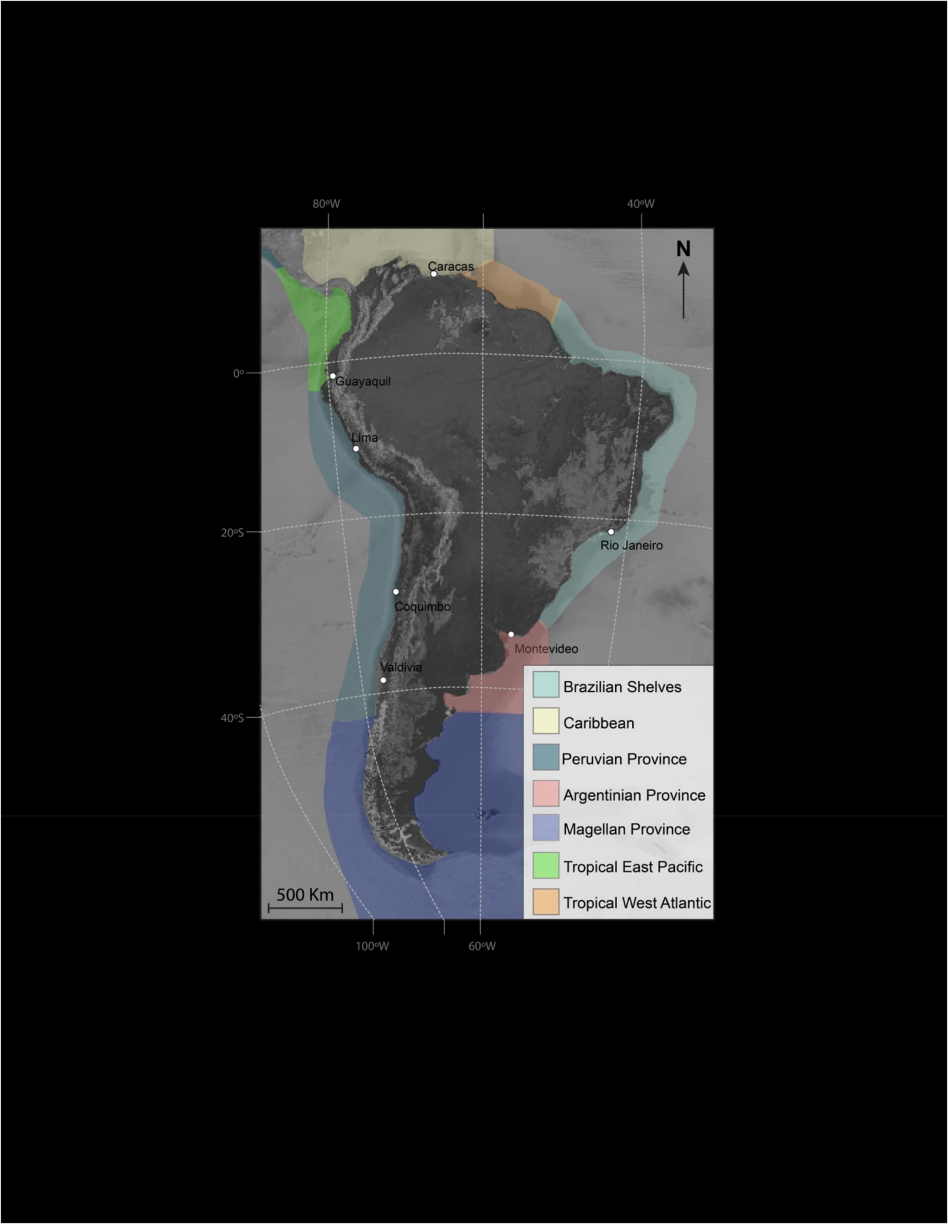
Map of the main South American Biogeographic marine areas. Modified from Miloslavish et al. (2011).

The temperate coasts of Peru and Chile, encompassing the Peruvian Province from about 6° to 42° S latitude (Figure 1), support one of the world's most productive marine ecosystems (Kämpf & Chapman, 2016). Observational and experimental investigations over the past 65 years have yielded a detailed empirical understanding of the zonation, composition, and interactions of intertidal and shallow subtidal bottom‐dwelling (benthic) species living along these coasts. Descriptive accounts of communities (Dayton, 1985; Guiler, 1959a, 1959b; Knox, 1960; Paine & Suchanek, 1983; Paine et al., 1985; Stotz et al., 2016) made some comparisons between Chile and other temperate coasts, but these and later works on predation and herbivory as well as explicitly biogeographic accounts (Blanchette et al., 2009; Cruz‐Motta et al., 2020; Thyrring & Harley, 2024) proceeded in the absence of a historical context. Insofar as these studies identified factors affecting the distribution of species, only present‐day conditions were considered. There was no consideration of how the biota was assembled, how extinctions and colonizations affected species composition and interactions, how past climatic changes affected the biota, and whether and to what extent the Peruvian Province functioned as a biogeographic refuge. As a result, the many highly unusual attributes of the biota of the Peruvian Province we recognize in this paper have remained largely unidentified. In particular, although the rocky intertidal ecosystems of the Peruvian Province superficially converge in structure on similar ecosystems along other coasts where upwelling is common (Blanchette et al., 2009), present‐day conditions do not suffice to account for the functional peculiarities we highlight here.

In parallel with research on the living biota, paleontologists have undertaken extensive studies of the rich fossil record of temperate western South America. With some notable exceptions (Grossmann et al., 2023; Kiel & Nielsen, 2010; Ochoa et al., 2021; Valdovinos et al., 2003), much of the descriptive work has concentrated on individual lineages, groups, or faunas. These important studies are essential for a comprehensive understanding of how the Peruvian Province's ecosystems and their components have weathered the many challenges of the last twenty‐five million years, but an integration with the findings on modern ecosystems has remained elusive.

In keeping with the view that an appreciation of history is essential to a full understanding of present‐day communities and ecosystems (Estes & Vermeij, 2022), we here synthesize evidence and insights from ecology, evolutionary studies, the geological and paleontological record, and comparisons with other ecosystems to evaluate how and to what extent the past has molded the composition, interactions, and future of the temperate nearshore ecosystems of western South America. Among other topics, we consider the role of extinctions, colonization of species from (and exchanges with) nearby regions and habitats, ecological replacements within functional categories (guilds) of species, and the unique features of ancient and modern communities along the Peruvian and Chilean coasts. Our aim is not simply to review what is known, but to highlight insights that previous studies did not provide.

## 2 GEOGRAPHIC AND CLIMATIC BACKGROUND

The Peruvian Province is generally considered warm‐temperate, climatically comparable with parts of New Zealand, southern Australia, southern Africa, Argentina, the North Atlantic, and North Pacific. Much of its coast is characterized by intense offshore upwelling during most years. Together with the high availability of iron, which is essential to diatom production, this makes the temperate coast of western South America by far the most planktonically productive region of the world (Kämpf & Chapman, 2016). The coast of Peru has perhaps the highest density of small pelagic fishes anywhere on Earth, permitting large populations of seabirds, which in turn produce vast deposits of phosphorus‐rich guano.

In contrast to most other temperate coasts, the coastline of the Peruvian Province faces the open ocean, with relatively few bays, lagoons, or other wave‐sheltered environments. Further south in Chile and elsewhere in the Magellan Province of southern Argentina, quiet‐water environments are more extensive, especially in the fjords that dissect the coast.

Every few years, when upwelling is strongly curtailed and nutrient‐rich waters are replaced by much warmer, more oxygenated and less nutrient‐rich waters during El Niño‐Southern Oscillation episodes (Escribano et al., 2004). Inshore primary productivity remains high even though the responsible phytoplankton comprises smaller‐celled species than during times of upwelling. The Humboldt Current System, which elicits upwelling along much of the coast, is thought to have been in place since at least the Late Eocene (Dunbar et al., 1990) or even the Paleocene (Keller et al., 1997). Before about 6 Ma, during the Late Miocene, the upwelled waters were warmer than they are today; thereafter, the component of cold Antarctic bottom water increased (Ibaraki, 1992; Kiel et al., 2023). Regardless of its source, upwelling was no less important during the warm Miocene and Pliocene than it is today (see Finger et al., 2007; Nielsen & Glotny, 2009; Ochoa et al., 2021; Ragaini et al., 2008).

Although long‐distance rafting of species on floating seaweeds maintains an intermittent biogeographic contact between western South America and New Zealand (Castilla & Guiñez, 2000; Fraser et al., 2011, 2022; Gordillo & Nielsen, 2013), much more extensive connections existed among southern land masses during the warm Late Oligocene to Early Miocene and the Early Pliocene (Beu et al., 1997; Casadío et al., 2010; Lauriat‐Rage et al., 2002). These connections are related to the formation of an early version of the Antarctic Circumpolar Current, made possible by West Antarctic rifting that broadened seaways between Antarctica and the other southern continents. Moreover, marine transgressions during warm intervals enabled many lineages to spread around the southern tip of South America (Encinas et al., 2014; Río et al., 2013).

## 3 METHODS

**FIGURE 2:**
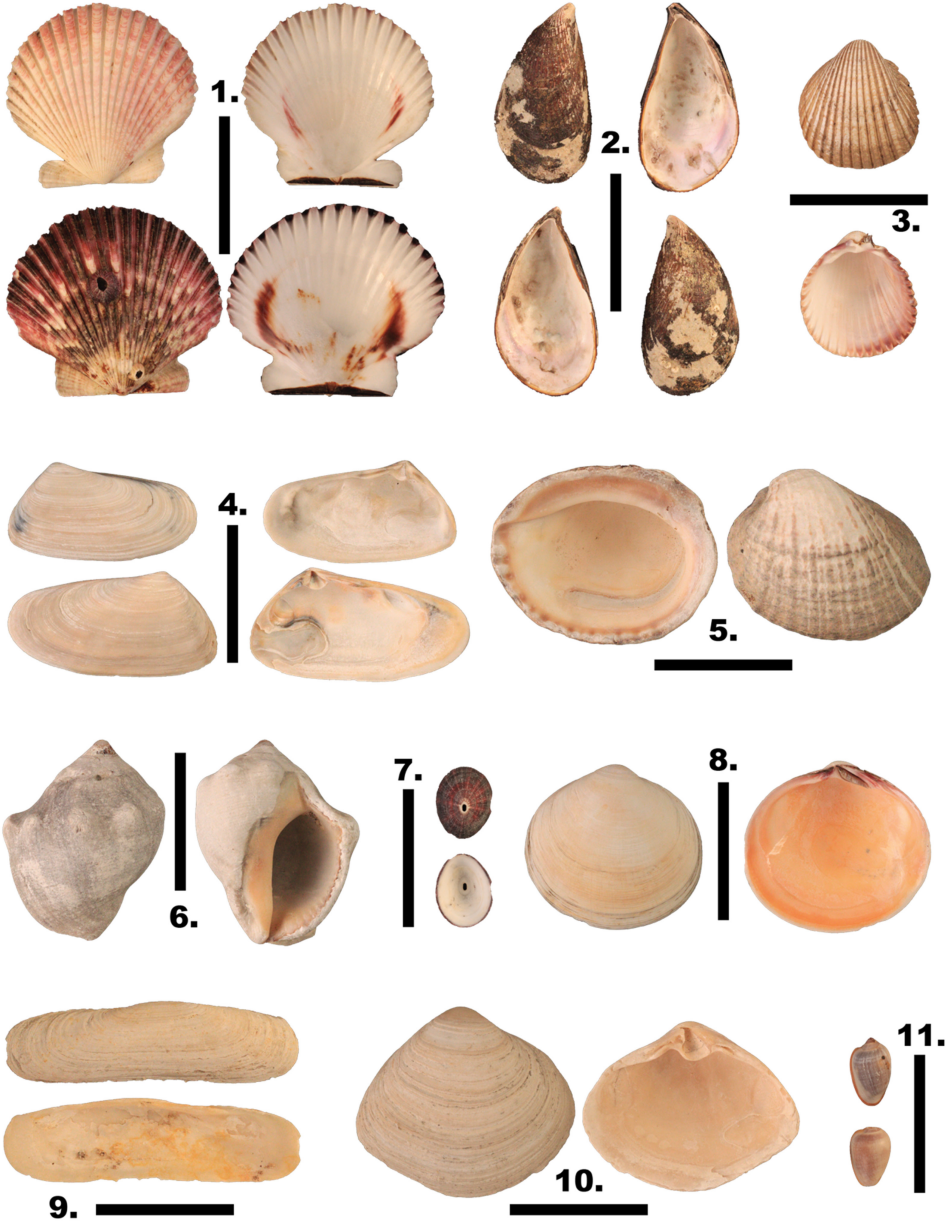
Some representative molluscan species of the Peruvian Province. 1. *Argopecten purpuratus* (13.89° S, 76.31° W); 2. *Aulacomya atra* (12.07° S, 77.16° W); 3. *Trachycardium procerum* (13.89° S, 76.31° W); 4. *Mesodesma donacium* (12.83° S, 76.54° W); 5. *Concholepas concholepas* (13.89° S, 76.31° W); 6. *Thaisella chocolata* (13.89° S, 76.31° W); 7. *Fissurella peruviana* (12.82° S, 76.55° W); 8. *Semele solida* (13.89° S, 76.31° W); 9. *Tagelus dombeii* (5.49° S, 81° W); 10. *Mulinia edulis* (17.42° S, 71.2° W); 11. *Prunum curtum* (13.89° S, 76.31° W). All specimens are housed at the Collection of Modern and Fossil Mollusks of the Peruvian Littoral, gathered by Luc Ortlieb and kept at the Universidad Peruana Cayetano Heredia (UPCH).

For the purposes of this paper, we consider the Peruvian Province to extend from 6° S to 42° S latitude. For fossil faunas we extend our coverage further south in Chile because the faunas in question represent conditions similar to those in the Peruvian Province today. We focus on benthic or benthically feeding organisms in shallow water (depth 100 m or less), especially organisms that are represented also by fossils (Figure 2). We excluded most commensal and parasitic species and organisms with maximum sizes less than 1 cm because they are taxonomically and paleontologically less well studied; as well as cold‐seep communities. Our temporal coverage encompasses the Neogene comprising the Miocene (23–5.4 Ma) and Pliocene (5.4–2.5 Ma) as well as the Pleistocene (2.5–0.01 Ma). Age assignments are corrected from original sources when necessary and follow the works of Kiel and Nielsen (2010), Ochoa et al. (2021), and for the southwest Atlantic Río et al. (2022) and Scasso et al. (2001).

We analyzed historical patterns not for the ecosystem as a whole, but for individual guilds – primary producers, herbivores, bioeroders, and several functionally different groups of predators – following Vermeij (2012b). For fossils, guild membership was determined from the known habits of morphologically similar extant species. Particular attention was paid to maximum body sizes of the largest members of guilds as documented in the taxonomic and paleontological literature (updated from Vermeij (2012b)). We focused on maximum body size because, all else being equal, large‐bodied individuals have greater per‐capita effects on other members of the ecosystem than smaller‐bodied species. Our emphasis on extreme size means that statistical tests based on size distributions are inapplicable. We frame our discussion as exploratory rather than hypothesis‐testing because we had no prior expectations (or hypotheses) about patterns in maximum body size over time.

## 4 ECOLOGICAL GUILDS AND THEIR HISTORY

### 4.1 Algae and seagrasses

In the modern flora of the Peruvian Province, Santelices and Meneses (2000) recorded 380 species of multicellular algae. Kelps of the genera *Lessonia* and *Macrocystis* in the Laminariales and the genus *Durvillaea* in the Fucales form extensive low intertidal and subtidal forests. Kelps colonized the Southern Hemisphere from the north no earlier than the Pliocene (Starko et al., 2019), whereas *Durvillaea*, a genus centered in New Zealand, diversified there twenty million years ago during the Early Miocene, based on molecular sequence data (Fraser et al., 2020). When *Durvillaea* might have reached South America remains unclear.

The Australasian seagrass genus *Heterozostera* is represented by two asexually reproducing clones, belonging to one or two species (Rothäusler et al., 2023; Smith et al., 2018). It occurs at two sites, between 27° and 30° S in central Chile. How and when *Heterozostera* arrived in Chile is not known. The genus is reported from the Pliocene (possibly Late Miocene) of southern Peru (Phillips et al., 1996).

### 4.2 Herbivores

The principal marine herbivores of the Peruvian Province are the echinoids *Loxechinus* and *Tetrapygus* (Navarrete et al., 2008; Vásquez & Buschmann, 1997); chitons (Camus et al., 2012; Sanhueza et al., 2008); limpet‐like gastropods (Aguilera, 2011; Aguilera et al., 2015; Aguilera & Navarrete, 2012; Aguilera, Navarrete, & Broitman, 2013; Aguilera, Valdivia, & Broitman, 2013); and fishes (*Aplodactylus*, *Girella*, *Scartichthys*, and *Sicyases*) (Ojeda & Muñoz, 1999; Paine & Palmer, 1978; Ruz et al., 2018). The abundance of herbivorous fishes in central Chile (20% of sampled individuals) is double that on the coast of California (Stepien, 1990), and is among the highest on any temperate coast. Endothermic marine herbivores are absent in the Peruvian Province; the kelp goose (genus *Chloephaga*) occasionally consumes algae, but it is restricted to the Magellan Province.

An unusual, terrestrially derived herbivore is the lizard *Mirolophus atacamensis*, which in the dry northern part of its range in the Atacama Desert regularly reeds on intertidal algae (Fariña et al., 2008). This is one of several cases of terrestrial herbivores in dry regions becoming more or less specialized to a diet of marine plants (Vermeij & Motani, 2018).

The Peruvian Province is unusual among temperate regions in lacking large limpets that individually catch and eat drift algae. Both *Loxechinus albus* and *Tetrapygus niger* catch drift algae, but *T. niger* does so only in aggregations (Dayton, 1985; Vásquez & Buschmann, 1997). The fact that none of the limpets exploit drift algae may be related both to their relatively small size (maximum length 135 mm in *Fissurella maxima*) and the limited ability of fissurellid keyhole limpets to clamp tightly. Chitons do adhere tenaciously to rocks and are large enough (200 mm in *Enoplochiton niger*), but they generally do not catch drift algae. Underexploitation of drifting algae also characterizes much of the North Atlantic guild of herbivores. The situation is quite different in the North Pacific and southern Africa, where haliotids (abalone) or large patellid limpets often specialize in feeding on such seaweeds.

The fossil record of molluscan herbivores in the Peruvian Province indicates that, with the exception of one Late Miocene species (*Cellana fuenzalidai*), the known Miocene and Pliocene limpets are smaller than their largest living counterparts. Fissurellids did not exceed a length of 60 mm during the Miocene and Pliocene (DeVries, 2008a; Nielsen et al., 2004), and the largest species of *Nacella* (*N. reicheae*, 80 mm) is also the oldest member of the genus, dating from the latest Oligocene of Peru (DeVries, 2008b). The largest Early Miocene limpet is *Scutelastra arayae* from Chile (93 mm) (Nielsen & Landau, 2014). The single large exception is *Cellana fuenzalidai* from the Late Miocene of northern Chile and southern Peru (Herm, 1969).

Two lineages of herbivorous marine mammals were present in the Peruvian Province during the Late Miocene to Early Pliocene. An unnamed small sirenian of the genus *Nanosiren* is known from fragmentary remains from the Late Miocene (Ochoa et al., 2021). The other is a lineage of edentate sloths of the genus *Thalassocnus*, known from a succession of species from the Late Miocene to Early Pliocene (8 to 4 Ma) in Peru and Chile (Amson et al., 2014, 2017; de los Arcos et al., 2017). The demise of most lagoons after the Pliocene eliminated most of the seagrasses on which these animals fed.

At least three genera of herbivorous fishes (*Aplodactylus*, *Girella*, and *Sicyases*) are known as Pliocene fossils in Chile (Oyanadel‐Urbina et al., 2021). The record for the blenny *Aplodactylus* is striking because kelps (*Lessonia trabeculata*), on which adults can feed, are thought to have colonized the Pacific coast of South America during the Pliocene.

### 4.3 Suspension‐feeders

As is true for temperate rocky shores elsewhere in the world, the most prominent fossilizable suspension‐feeders in the intertidal zone of the Peruvian Province are mussels (mytilid bivalves) and balanomorph barnacles. Mussels are represented by six genera, each with a single species: the small upper‐shore *Perumytilus*, the small mid‐shore *Hormomya* (often referred to as *Brachidontes*) and *Semimytilus*, and the much larger low‐shore *Aulacomya* (*A. atra*, 154 mm), *Choromytilus* (*C. chorus*, 300 mm), and *Mytilus* (*M. chilensis*, 78 mm) (Valentich‐Scott et al., 2020). The huge size of *C. chorus* is reached in the fjords of southern Chile rather than in the Peruvian Province (Vermeij, 2012b).

Sessile barnacles in the Peruvian Province belong to seven genera: *Austromegabalanus*, *Balanus*, *Elminius*, *Jehlius*, *Notobalanus*, *Notochthamalus*, and *Verruca* (Buckeridge & Newman, 2010; Foster & Newman, 1987; Newman, 1979). At a test height of up to 30 cm, the subtidal *A. psittacus* is one of the world's largest barnacles (Buckeridge, 2015). Barnacles have been prominent members of hard‐bottom communities since at least the Early Miocene (represented by *Austromegabalanus* and the extinct *Perumegabalanus*) and Pliocene (*Austromegabalanus*, *Balanus*, *Tesseropora*, and *Verruca*) (Carriol et al., 1987; Carriol & Schneider, 2013; Coletti et al., 2019; Collareta et al., 2019; Nielsen, 2009).

In the low intertidal and shallow subtidal zones, suspension‐feeding calyptraeid slipper limpets are prominent, large, and diverse. Reaching a diameter of 100 mm, species of *Trochita* grip the rock tightly enough to enable kelps to grow on them. Other genera include *Crepidula* (hermit crab‐associated species), *Crucibulum*, and *Trochita* (Pastorino & Urteaga, 2012; Véliz et al., 2001, 2003, 2012). The genera *Trochita* and *Crepipatella* are known in the Peruvian Province back to at least the Early Miocene (Kiel & Nielsen, 2010). In the shallow subtidal zone, the solitary ascidian genus *Pyura* is represented in the Peruvian Province by one native species (*P. chilensis*) and one species (*P. praeputialis*) from Australia that was apparently introduced by humans and has remained more or less confined to the Bay of Antofagasta in northern Chile (see Castilla et al., 2002, 2004, 2014; Paine & Suchanek, 1983; Rius et al., 2017; Stotz et al., 2016). There is no known fossil record of *Pyura*, but the genus is native only to the Southern Hemisphere, where it can achieve enormous biomass (Rius et al., 2017).

On soft bottoms, fossilizable suspension‐feeders are represented by bivalves. These include fast‐burrowing razor clams of the genera *Ensis*, *Ensisolen*, and *Tagelus*, as well as slower‐burrowing venerids (*Eurhomalea*, *Ameghinomya*, *Retrotapes*), semelids (*Semele*), and mactrids (*Mulinia*). The largest member of this second group in the Peruvian Province is *Eurhomalea rufa* (length 115 mm) (Valentich‐Scott et al., 2020). Maximum sizes were greater during the Pliocene, with southern specimens of the still living venerid *Dosinia ponderosa* (now confined to the tropical Panamic Province) reaching 140 mm (Herm, 1969). These Pliocene and Recent species are all larger than any venerid in the Navidad Formation or equivalents of the Early Miocene of Chile (maximum length 97.5 mm in *Chione rodulfi*) (Frassinetti & Covacevich, 1993).

An important component of the epifauna on unconsolidated bottoms throughout the world's temperate zones comprise scallops of the bivalve family Pectinidae. In the modern Peruvian Province, this guild is represented by the large (140 mm) *Argopecten purpuratus*, a species of tropical origin extending from Peru to northern Chile, especially during warm years. No other pectinid, large or small, currently lives in the Peruvian Province. Other Chilean species either live in deep water or occur further south in the Magellan Province (Rosenfeld et al., 2021).

Fossil pectinids in the Peruvian Province achieved maximum sizes close to or a little smaller than the living *A. purpuratus*. The Pliocene *Dietotenhosen remondi* had a diameter of 120 mm, somewhat smaller than *“Chlamys” simpsoni* (138 mm) (Herm, 1969; Santelli & del Río, 2019a). The Early Miocene *Zygochlamys geminatus* (120 mm) is again of comparable size (Santelli & del Río, 2019b).

None of the pectinids living on the continental shelf of Argentina is large, but during the Miocene scallops in the southwestern Atlantic achieved sizes comparable with large pectinids in Chile. These include the Early Miocene *Zygochlamys geminatus* (120 mm) and *Reticulochlamys borjasensis* (134 mm) and the Late Miocene *Moirechlamys actinodes* (136 mm) (del Río, 2004; Santelli & del Río, 2019a, 2019b).

Bivalves of the family Isognomonidae are byssally attached members of the epifauna, mostly found in shallow tropical waters. The large‐bodied genus *Neopanis* is represented in the Early Miocene of New Zealand, Chile, and perhaps Argentina (Beu, 2004). The last isognomonid in the Peruvian Province is *Isognomon gaudichaudi*, a Pliocene species that reached a maximum dimension of at least 279 mm (Griffin & Nielsen, 2008), making it the largest known bivalve in the Chilean Pliocene.

Four guilds of suspension‐feeding bivalves are very poorly represented in the extant Peruvian Province but were prominent members during the Miocene and Pliocene. The first comprises larger oysters (Ostreidae). In the modern fauna, there are two oyster‐like bivalves, the true oyster *Ostrea chilensis* (length up to 85 mm) in quiet waters and the open‐coast chamid *Chama pellucida* (70 mm) (Valentich‐Scott et al., 2020). In Pliocene strata at Coquimbo, Chile, the large oyster *“Crassostrea” transitoria* reached a length of at least 217 mm (Griffin & Nielsen, 2008). Since the demise of this species, the guild of cemented bivalves was filled by species that appear to have colonized the Peruvian Province recently. *O. chilensis* perhaps from New Zealand (Ó Foighil et al., 1999; but see Beu, 2006 for the argument that the lineage of *Ostrea* reached New Zealand from South America during the Late Miocene) and *C. pellucida* from the northeastern Pacific (Bernard, 1976). Very large oysters existed in the Miocene through Early Pleistocene of Peru, but these have not been studied.

The Argentinian fauna likewise supports only small oysters (Shilts et al., 2007). Vast beds of large oysters are known from the Early Miocene and Late Miocene of Argentina (Cuitiño et al., 2013; Parras & Casadío, 2006), comprising not only species of *“Crassostrea”*, but also *Cubitostrea alvarezi*. *“Crassostrea” hatcheri* in the Monte León Formation (Early Miocene) reached a length of at least 250 mm and a valve thickness of 70 mm (Parras & Casadío, 2006). In the Late Miocene Puerto Madryn Formation *“Crassostrea” patagonica* reached 172 mm (Griffin & Nielsen, 2008).

The second guild comprises slow‐burrowing, nonsiphonate bivalves in unconsolidated sediments, especially members of the Arcidae, Cucullaeidae, Glycymerididae, Carditidae, and Crassatellidae. Today this guild is represented in the Peruvian Province by two glycymeridids (maximum length 56 mm in *Glycymeris intermedia*) and by the small carditids of the genus *Cyclocardia* (Güller & Zelaya, 2013; Valentich‐Scott et al., 2020). The nonsiphonate burrowing guild was much richer in species, and attained much larger body sizes, during the Early Miocene to Pliocene interval. In the Navidad Formation (Early Miocene) of Chile, *Cucullaea chilensis* exceeded a length of 119 mm (Frassinetti & Covacevich, 1993) and *Glycymeris ibariformis* (more than 100 mm long). The largest Pliocene species of this guild is *“Anadara” chilensis* (73 mm) (Nielsen, 2013). In the Early Miocene of Peru, several species of the crassatellid genus *Tilicrassatella* reached a length of 100 mm (DeVries, 2016b).

In most parts of the world, cockles (Cardiidae) are well represented by highly active shallow burrowers, which often can also leap or swim when in peril. This was certainly the case in the Early Miocene of Chile. Besides the small‐bodied genus *Pratulum*, represented by two species, and the somewhat larger *“Trachycardium” multiradiatum* (length 86 mm), there were two species of the Southern‐Hemisphere genus *Lahillia*, including the large *L. angulata* (146 mm). (Frassinetti & Covacevich, 1993; Griffin & Nielsen, 2008; Rojas & Nielsen, 2020). *Mexicardia domeykoana*, an extinct temperate member of an otherwise tropical genus, reached a length of 128 mm during the Pliocene in Chile (Herm, 1969). There is no extant cardiid in the Peruvian Province.

A similar decline in diversity and maximum shell size affected this guild in the southwestern Atlantic. During the Early Miocene, at least five species approached or exceeded a length of 100 mm: *Glycymeris pseudocuevensis* (116 mm), *Glycymerita cuevensis* (128 mm), *G. camaronesia* (104 mm), the cucullaeid *Monteleonia alta* (94 mm), and the carditid *Neovenericor austroplata* (120 mm) (del Río, 1992; del Río & Camacho, 1998; Pérez et al., 2017). In the Late Miocene Puerto Madryn Formation in Chubut Province, *Glycymeris magna* reached a length of 118 mm (del Río & Camacho, 1998). As in the southeastern Pacific, the modern shallow‐water fauna of temperate Argentina contains only small nonsiphonate burrowing bivalves, all belonging to Carditidae.

A unique feature of the Peruvian Province is the absence of deep‐burrowing, sedentary, siphonate bivalves. The hiatellid *Panopea coquimbensis* which became extinct in southern Peru during the Early Pliocene (DeVries & Frassinetti, 2003), was the last representative of this functional group in the province. The mactrid *Darina solenoides* represents this guild in the Magellan Province and in Argentina. In temperate Argentina this species is joined by species of *Panopea* and *Scobinopholas*.

### 4.4 Bioeroding bivalves

Bivalves boring into rocks or shells are well represented in the extant fauna of the Peruvian Province. Valentich‐Scott et al. (2020) recorded eight species in four families with wide ranges in the Peruvian Province; three in Veneridae (Petricolinae), two in Pholadidae, two in Mytilidae, and one in Hiatellidae (see also Zelaya & Güller, 2023). Several are very large, indicating deep bioerosion; *Pholas chiloensis*, 131 mm; *Leiosolenus peruvianus*, 76 mm; and *Petricola rugosa*, 50 mm. Little can be said about the fossil record of this guild in the Peruvian Province because fossils have not been well studied.

### 4.5 Benthic predators

Among the guilds of bottom‐feeding predators of hard‐shelled prey in the Peruvian Province are one mammal (the sea otter *Lontra felina*) several birds, fishes, and various gastropods, crabs, and sea stars. The sea otter occurs on exposed rocky shores and forages on mollusks (Castilla & Bahamondes, 1979). Among birds, the most important are two species of *Cinclodes*, songbirds that feed on upper‐shore mollusks (Hockey et al., 1987), the oystercatcher *Haematopus ater* (Hockey et al., 1987; Pacheco & Castilla, 2000), and the surfbird *Aphriza virgata* (Navarro et al., 1989). *Lontra felina* has a Late Pleistocene origin in western South America (Vianna et al., 2010, 2011). Little can be said about the history of the birds because relevant fossils are unknown.

Fishes that include significant numbers of shell‐bearing prey in their diets include *Semicossyphus maculatus*, *Cheilodactylus variegatus*, *Pinguipes chilensis*, *Anisotremus scapularis*, and *Hemilutjanus microphthalmos* as well as the cartilaginous fishes *Schroederichthys chilensis* and *Pseudobatos planiceps* (González‐Pestana et al., 2021; Medina et al., 2004). Although species composition has changed over time, this guild was well represented from the Early Miocene onward (Chávez‐Hoffmeister & Villafaña, 2023; Oyanadel‐Urbina et al., 2021).

During the Late Miocene and Early Pliocene, the guild of bottom‐feeding marine mammals comprised the phocine seal genus *Hadrokirus*, the walrus‐like delphinid cetacean *Odobenocetops*, and a large walrus (Amson & de Muizon, 2014; de Muizon & Domning, 2002; Ochoa et al., 2021). None of the extant otariid seals in the Peruvian Province has diets of hard‐shelled prey as these fossil groups did.

Among other non‐molluscan benthic predators, brachyuran crabs on both hard and unconsolidated bottoms are likely important but not well studied. Known or probable mollusk‐eating crabs include *Homalaspis plana* (Morales & Antezana, 1983) and cancrids of the genera *Cancer*, *Metacarcinus*, and *Romaleon*. The discovery of a species of *Romaleon* (*R. parspinosus*) in the Puerto Madryn Formation (Late Miocene) of Argentina (Casadío et al., 2005) indicates that cancrids have been in southern South America since at least that time, but little else can be said about the fossil record of predatory crabs in the Peruvian Province. No cancrids have yet been recorded from the Early Miocene of Chile (Feldmann et al., 2005, 2010).

Predatory sea stars in the Peruvian Province belong to the genera *Heliaster*, *Meyenaster*, and *Stichaster* (Dayton et al., 1977; Escobar et al., 2018; Escobar & Navarrete, 2011; Paine et al., 1985; Tokeshi et al., 1989). They feed on mollusks, barnacles, and the ascidian *Pyura* (see also Manzur et al., 2014; Manzur & Navarrete, 2011). The largely subtidal *Meyenaster gelatinosus* can feed on and controls populations of *Heliaster helianthus*, whose distribution is largely intertidal where the two species overlap (Gaymer & Himmelman, 2008).

A notable, though not unique, feature of the intertidal fauna of temperate western South America is the high prevalence of black or dark‐colored species (DeVries, 2006; Vermeij, 2020). Black shells are conspicuous in the gastropods *Diloma nigerrima*, *Fissurella nigra*, *Chlorostoma atrum*, and *Prisogaster niger*; the chiton *Enoplochiton niger*; the mussel *Perumytilus purpuratus*; and the echinoid *Tetrapygus niger*. These species are abundant, many reaching the middle and upper zones of the rocky intertidal. DeVries (2006, 2007c) showed that black shells in *Chlorostoma* and *Prisogaster* evolved from lighter‐colored congeners during the Late Pliocene. Keyhole limpets of the genus *Fissurella* in South America are known only from the Pliocene onward (DeVries, 2008a), and *Tetrapygus* may be no older than the Pleistocene (Courville et al., 2023). Citing evidence that some sea stars can sense the difference between light and dark, Vermeij (2020) speculated that the dark shells might be visually cryptic to visually hunting sea stars such as *Heliaster*, *Meyenaster*, and *Stichaster*, and perhaps birds. The west coast of temperate South America shares with the Pacific coast of North America the presence of common dark‐colored gastropods and of middle to upper intertidal predatory sea stars, which are known to be important predators of mollusks and sea urchins. On other temperate rocky shores, dark gastropods and predatory sea stars are either absent or are limited to the low intertidal zone or subtidal habitats.

It is not currently known when sea stars first occupied the upper reaches of the intertidal zone in temperate western South America, but they may have done so relatively recently. Outside the Peruvian Province, *Heliaster* is represented by one living species in the Gulf of California and by a Pliocene species in Florida; it could therefore be part of a larger cohort of lineages that colonized Peru and Chile after the Pliocene extinctions. The genus *Stichaster* occurs in New Zealand and could be part of a group of lineages that colonized Chile by open‐ocean dispersal on algal rafts during the Pleistocene. None of the generally deep‐water echinoderms recorded from the Navidad Formation (Early Miocene) of Chile belongs to any of the Recent predatory seastar genera in the Peruvian Province (Kutscher et al., 2004).

Muricid gastropods are important predators on both hard and soft bottoms in the Peruvian Province. Most drill a hole through the external skeletons of mollusks and barnacles or through the outer wall of tunicates. Their role on rocky bottoms and subtidal sand in Chile has been studied by Martinelli et al. (2022).

Another unique attribute of the shallow‐water fauna of temperate western South America is the high incidence of predatory gastropods with a labral tooth, a ventrally oriented projection on the anterior sector of the shell's outer lip. This feature is associated with faster predation on mussels and barnacles compared with drilling through the shell. In the extant fauna of the Peruvian Province, three of ten shallow‐water muricid species (30%) have a labral tooth (two teeth in *Concholepas concholepas*). This incidence is higher than in any other regional muricid fauna (Martinelli et al., 2022; Vermeij, 2001). Labral teeth were even more common during the Miocene and Pliocene. Of four recorded muricids in the Early Miocene, three (75%) have a labral tooth. In addition, *Testallium cepa* (Pseudolividae) and *Macron vermeiji* (Buccinoidea) also developed a labral tooth. The incidence remained exceptionally high during the Early Pliocene (five of eleven muricid species, 45%, not counting the tooth in *Testallium escalonia*). In the rich muricid fauna of sixteen species during the Late Pliocene, eight (50%) retained or evolved a labral tooth.

In the southwestern Atlantic north of the Magellan Province, the modern temperate fauna contains no muricid or any other gastropod with a labral tooth. By contrast, at least three Early Miocene muricids had such a tooth: *Entacanthus monoceros*, *Argenthina emilyae*, and *“Trophon” boggsi* (see Griffin & Pastorino, 2005; Herbert & del Río, 2005; von Ihering, 1907).

In the modern fauna, the guild of large predatory gastropods on unconsolidated bottoms is poorly represented, with only three species in three families. The ocenebrine muricid *Chorus giganteus* reaches a length of 130 mm, about the same length as the tudiclid buccinoidean *Aeneator castillai* (128 mm) (DeVries, 1997; Osorio & Ramajo, 2007). The third species, the volutid *Miomelon alarconi*, attains a length of 90 mm, but this species lives in much deeper water (McLean & Andrade, 1982). Early Miocene members of this guild were much larger and more diverse. The largest species in Chile was the volutid *Adelomelon alta* (195 mm), but other species of *Adelomelon* and the extinct genus *Palaeomelon* were also large (Nielsen & Frassinetti, 2007b). The Early Miocene Peruvian buccinoidean genus *Misifulgur*, represented by two species, reached lengths of 110–120 mm. The genus *Chorus*, with a length of up to 90 mm, entered this guild in Peru during the Late Miocene (DeVries, 1997).

In contrast to the southeastern Pacific, large volutids remain prominent predators on the continental shelf of Argentina, represented by the genera *Adelomelon*, *Odontocymbiola*, *Pachycymbiola*, and *Zidona*. The largest of these, *A. beckii*, reaches a length of 500 mm and is one of the world's largest volutids. Volutids were already large during the Early Miocene, as indicated by *A. pilsbryi* (230 mm) in the Monte Leon Formation (del Río & Martínez, 2006).

Naticid gastropods are abundant drilling predators almost everywhere in the world, but in the Peruvian Province there is a single species, *Sinum cymba*, which extends from tropical Ecuador south to Caldera in northern Chile (Marincovich Jr., 1977). The group was represented by several genera in the Early Miocene of Chile, including the large *Magnatica subsolida* (42 mm), and by a few species in the Pliocene.

### 4.6 Limpet‐like gastropods

No rocky intertidal gastropod fauna has as high a proportion of limpet‐like species as does that of central Chile (27 of 58 species, 47%, based on Aldea & Valdovinos, 2005; see Vermeij, 2017). The proportion in the Magellan fauna of southern Chile is nearly as high (12 of 30 species, 40%, based on Reid & Osorio, 2000). In temperate western South America, the limpet form is represented by members of the Calyptraeidae, Fissurellidae, Lottiidae, Muricidae (*Concholepas*), Nacellidae, and Siphonariidae.

## 5 LARGE‐SCALE PROCESSES AND EVENTS

### 5.1 Latitudinal diversity gradients

A highly unusual biogeographic feature of the biota of temperate western South America is what has been described as a reversed or humped latitudinal gradient in species diversity. In contrast to the prevailing (but not universal) pattern of increasing species from high to low latitudes (that is, toward the warmer tropics), the fauna and flora of the cold Magellan Province of southern South America are richer in species than is the Peruvian Province. This pattern has been documented for seaweeds (Santelices & Meneses, 2000), mollusks (Kiel & Nielsen, 2010; Valdovinos et al., 2003), echinoids (Vásquez & Buschmann, 1997), and polychaete annelids (Moreno et al., 2021). The highest diversity in this region occurs around the Chiloé archipelago, at about 42° S. The same pattern is seen in the diversity of functional groups of mollusks (Grossmann et al., 2023), whereas during the Early Miocene, both the diversity of species and the diversity of functional categories followed the more conventional poleward decrease (Grossmann et al., 2023).

According to Kiel and Nielsen (2010), this reversed latitudinal gradient originated during the Pleistocene, after the extinctions of the Pliocene. They attributed the establishment of this pattern to the great post‐Pliocene reduction in bays and lagoons along the coast of the Peruvian Province (see also Dunbar et al., 1990). Shores exposed to the full force of the temperate South Pacific would always have been less rich in species than the quieter waters of embayments. In southern Chile, south of the Peruvian Province, many quiet‐water habitats remain in fjords and bays, which owing to rapid movements of nutrient‐rich tidal waters support a highly productive, species‐rich biota (see also Vermeij, 2012b). A drastic post‐Pliocene drop in sea water temperature might explain the high magnitude of extinction but not the reversed diversity gradient, because diversity is higher in the Magellan Province than in the warmer waters further north. An episodically very shallow oxygen minimum zone (depth as little as 25 to 50 m) in Peru and northern Chile (Morales et al., 1996) that would certainly limit the occurrence of sediment‐dwelling species but whether this is the cause or the consequence of periodically high mortality remains an open question. The selective survival of deposit‐feeding bivalves, which generally tolerate low oxygen concentrations, is consistent with the role of dysoxia in restricting the diversity of sediment‐dwellers (Rivadeneira & Marquet, 2007).

Whether the loss of species‐rich habitats is the whole story to explain the reversed latitudinal gradient is unclear. Large bays and an extensive inland sea in California were largely eliminated after the Pliocene as well, but here there is no evidence for a reversed gradient, nor indeed of the high magnitude of extinction that characterizes the Peruvian Province. In the northwestern Atlantic, lagoons, bays, and unconsolidated sediments on the continental shelf have remained prominent features of the coastal marine topography; yet this area witnessed severe extinction after the Pliocene without the diversity gradient undergoing a regional reversal (Vermeij et al., 2008; Vermeij & Ruch, 2018). Perhaps the additional challenge of severe, frequent climatic oscillations related to El Niño that affects the Peruvian Province is a contributing factor.

### 5.2 Origins of the Peruvian Province biota

Much of the biota in the extant Peruvian Province has deep Southern Hemisphere roots extending to the Miocene and often much earlier. Despite the fact that conditions were warmer in the Peruvian Province during the Miocene and Pliocene than they are today, these old southern elements are fundamentally temperate groups unrelated to the tropical lineages that existed further north. In other words, the fauna and flora of the Peruvian Province had far more in common with that of temperate Australasia than with tropical America (Beu et al., 1997; Casadío et al., 2010).

In the rocky intertidal and shallow subtidal zones, ancient southern roots have been phylogenetically or paleontologically documented for the brown‐algal genus *Durvillaea* (Fraser et al., 2020), the ascidian genus *Pyura* (Rius et al., 2017), the sea‐star genus *Stichaster* (Mah & Foltz, 2011), the *Acanthina* clade of ocenebrine muricid gastropods (Barco et al., 2017), the rapanine muricid genus *Concholepas* (Claremont et al., 2013; DeVries, 1995), the littorinid gastropod genus *Austrolittorina* (Reid & Williams, 2004), the trochoidean gastropod *Diloma* (Donald et al., 2005), the patellogastropod genera *Nacella* and *Scurria* (DeVries, 2008b; Espoz et al., 2004; González‐Wevar et al., 2011, 2019; Lindberg, 1988; Nakano & Ozawa, 2007), the tudiclid buccinoidean whelk *Aeneator* (Kantor et al., 2022), the endolithic hiatellid bivalve *Hiatella* (Zelaya & Güller, 2023), four genera of mussels (*Aulacomya*, *Choromytilus*, *Perumytilus*, and *Semimytilus*) (Beu et al., 1997; Trovant et al., 2015), the keyhole limpet *Fissurella* (DeVries, 2008a), and almost all the barnacles (Buckeridge, 2015; Buckeridge & Newman, 2010; Newman, 1979). Several lineages on unconsolidated bottoms also have southern temperate origins, including venerid bivalves (*Ameghinomya*, *Eurhomalea*, and *Retrotapes*) (Alvarez, 2019; Pérez et al., 2013), the turritellid gastropod *Incatella* (DeVries, 2007a), zidonine and odontocymbioline volutids (Nielsen & Frassinetti, 2007b), and the seagrass *Heterozostera* (Kuo, 2005). As discussed further below, most extinct groups in the Miocene and Pliocene of Peru and Chile also have southern affinities.

There are, however, important north‐temperate elements that have colonized the Peruvian Province. These include the kelps (Starko et al., 2019), otariid seals (Benites‐Palomino et al., 2022; Valenzuela‐Toro et al., 2016; Yonegawa et al., 2009), cancrid crabs (Schweitzer & Feldmann, 2000), the rapanine muricid *Stramonita* (DeVries, 2007b), teguline trochoideans (DeVries, 2007c), the lottiid limpet *Lottia orbignyi* (Espoz et al., 2004), and the mussel *Mytilus* (Gaitán‐Espitia et al., 2016; Gérard et al., 2008; Hilbish et al., 2000). Unlike the ancient southern element, most of the northern lineages colonized the Peruvian Province during or after the Pliocene; only the cancrids extend back to the Miocene (see also Casadío et al., 2005; Robin et al., 2018).

Two recent additions to the make‐up of the Peruvian Province fauna are noteworthy. One is the Late Pleistocene origin of the sea otter *Lontra felina* from a Patagonian lineage (de Ferran et al., 2022; Vianna et al., 2010, 2011). The second is the Late Pleistocene to Holocene arrival of humans, which have of course had a profound effect on the entire biota (reviewed in Moreno, 2001). As is true in other regions, all human‐exploited molluscan species in the Peruvian Province exhibit a reduction in maximum body size owing to the human preference for large prey (Vermeij, 2012a). This is a highly unusual trend for a predator.

### 5.3 Evolutionary innovation

Vermeij (2018) identified ten innovations in thirteen lineages during the Miocene to Recent interval in southern South American animals. Only seven of these in ten clades, however, evolved in situ in the Peruvian Province: algal grazing by *Sicyases*, herbivory in *Thalassocnus* (marine sloth), predation on hard‐shelled prey by three lineages of mammals (*Hadrokirus*, *Lontra*, and *Odobenocetops*), rock excavation by scurriine lottiid limpets, kelp‐stipe excavation by *Scurria scurra*, labral‐tooth evolution in *Concholepas* and the *Acanthina* clade of muricids, and the limpet form in *Concholepas*. The other innovations evolved further south in the Magellan Province. To the list of Peruvian Province innovations must be added the feeding by songbirds of the genus *Cinclodes* on intertidal mollusks (Vermeij & Motani, 2018). The mammalian innovations all appeared in lineages that, with the exception of *Lontra*, are now extinct.

### 5.4 Extinction and refuges

The temperate west coast of South America witnessed high magnitudes of extinction during the Miocene and Pliocene. Of ninety‐one molluscan genera then recognized in the Navidad Formation (Early Miocene) of Chile (Finger et al., 2007), 12% became extinct globally. Our compilation identifies sixteen molluscan genera from the Early Miocene of Peru and Chile that became globally extinct: *Ameranella* (Beu, 2010), *Austrocominella* (Kiel & Nielsen, 2010), *Austroimbricaria* (Nielsen & Ampuero, 2020), *Austrombus* (Nielsen, 2005), *Carhuaspina* (DeVries, 2005; see also, Kiel & Nielsen, 2010), *Crassicymatium* (Beu, 2010), *Fagnastesia* (Nielsen et al., 2004), *Hemichenopus* (Nielsen & Encinas, 2014), *Intitectonica* (Nielsen & Frassinetti, 2007a), *Ipunina* (Nielsen & Frasslnetti, 2008), *Matanziella* (Frassinetti & Covacevich, 1993), *Misifulgur* (DeVries, 2016a), *Palaeomelon* (Nielsen & Frassinetti, 2007b), *Struthiochenopus* (Nielsen, 2005), *Tactilispira* (DeVries, 2005), and *Tilicrassatella* (DeVries, 2016b).

Additional extinct genera in the Navidad Formation disappeared later elsewhere (see below). Non‐molluscan genera that became extinct after the Early Miocene in Chile and Peru include the barnacle *Perumegabalanus* (Coletti et al., 2019), the fossulasterid seastar *Philipaster* (Kutscher et al., 2004), the crabs *Proterocarcinus* (Macropipidae) and *Pirulella* (Atelecyclidae) (Feldmann et al., 2005), and four genera (out of 44 recorded from otoliths) or bony fishes: *Karrerichthys*, *Navidadichthys*, *Paracarapus*, and *Sirembola* (Schwarzhans & Nielsen, 2021).

We identify at least 25 genera of Early Miocene mollusks from Peru and Chile that are found today only in tropical waters or are widespread on tropical and temperate coasts. The following 17 genera from the Early Miocene survive in the Peruvian Province today: *Acanthina*, *Aeneator*, *Ameghinomya*, *Choromytilus*, *Crepipatella*, *Diloma*, *Ensisolen*, *Felicioliva*, *Gemmula*, *Incatella*, *Miomelon*, *Nacella*, *Ptychosyrinx*, *Retrotapes*, *Sinum*, *Stramonita*, and *Trochita*. It is remarkable that only two of these genera (*Aeneator* and *Diloma*) also occur in New Zealand; *Nacella* is widely distributed in sub‐Antarctic and Antarctic waters, and *Crepipatella* and *Trochita* are known from various upwelling sites including southern Africa.

Extinctions during the Late Pliocene were also severe. Our compilation shows five molluscan genera that became globally extinct: the muricid genus *Herminespina* (DeVries & Vermeij, 1997), the scallops *Dietotenhosen* and *Ckaraotippur* (Santelli & del Río, 2019a), the pseudolivid gastropod *Testallium* (Vermeij & DeVries, 1997), and the venerid bivalve *Austrocallista* (Alvarez et al., 2020). Some 42% of vertebrate marine genera, including all bottom‐feeding marine mammals, the fish‐eating ocean‐going bony‐toothed bird *Pelagornis*, two fish‐eating marine crocodylians, and the apex predator *Otodus megalodon* (the megatooth shark) also disappeared (Mayr & Rubilar‐Rogers, 2010; Ochoa et al., 2021; Salas‐Gismondi et al., 2022; Villafaña & Rivadeneira, 2014). Species‐level losses range from 89% for Peruvian mollusks (DeVries, 2005) and 61%–76% for specific sites in Peru and Chile (Rivadeneira & Nielsen, 2017). By contrast, only two of twenty‐three Pleistocene mollusk species from Tubul, Chile (8.7%) became extinct (Nielsen & Valdovinos, 2008).

Two regions have served as geographic refuges for temperate lineages that became extinct in the Peruvian Province. At least seventeen mollusks became restricted since the Early Miocene to Australasia: *Amalda*, *Austrotoma*, *Awheturris*, *Bedeva*, *Cirsotrema* (large species), *Fusiguraleus*, ?*Kaotoa*, *Lamprodomina*, *Magnatica*, *Marshallena*, *Neopanis*, *Lisanerita*, *Penion*, *Pratulum*, *Xymene*, *Xymenella*, and *Zeacuminia* (Beu et al., 1997; Rojas & Nielsen, 2020; Vermeij et al., 2009). At least seven of these taxa also became extinct in New Zealand but did so during or after the Pliocene: *Austrotoma*, *Awheturris*, *Lamprodomina*, *Magnatica*, *Marshallena*, *Neopanis*, and *Zeacuminia* (see Beu, 2011).

The other refuge is the coast of Argentina in the southwestern Atlantic. Early Miocene genera that became regionally extinct in the Peruvian Province but that survive in Argentina are *Adelomelon*, *Austrotoma*, *Olivancillaria*, and *Pachycymbiola* (Beu, 2011; Nielsen, 2004; Nielsen & Frassinetti, 2007b). Three genera became restricted to the Southern Hemisphere in the southwestern Atlantic after the Pliocene or Early Pleistocene: *Eucallista*, *Euspira*, and *Panopea*; and a fourth (*Sassia kampyla*) remains widely distributed in the Southern Hemisphere but no longer lives in Chile (Beu, 2010). All Argentina‐restricted molluscan taxa are characteristic of unconsolidated bottoms; and all except *Austrotoma* and *Panopea* are unknown from New Zealand. In addition, the lineage leading from the extinct Miocene southeastern Pacific Pontoporiid dolphin genus *Brachydelphis* to the extant *Pontoporia* became restricted to the Atlantic coast of southern Brazil to northern Argentina (Lambert & de Muizon, 2013).

Is the Peruvian Province a biogeographic refuge? Only four marginal cases of restriction to the Peruvian Province from a broader distribution can be identified, but all come with caveats as discussed below. Importantly, no lineage known from the fossil record of New Zealand or Argentina has become restricted to the Peruvian Province.

One case of restriction involves the barnacle genus *Notobalanus*, represented in the Peruvian Province today by the abundant *N. flosculus*. The only occurrence outside the Peruvian Province is a record of *N. flosculus* from the Early to Middle Miocene of Kerguelen Island in the southern Indian Ocean (Carriol et al., 1992; Lauriat‐Rage et al., 2002). To our knowledge, *Notobalanus* has not been reported as a fossil from either New Zealand or Argentina.

The second case is the large limpet‐like muricid *Concholepas concholepas*, the single recent representative of a western South American lineage dating back to the Middle Miocene (DeVries, 1995). Kensley (1985) recorded several specimens of this species from the Pleistocene of the west coast of South Africa, where it does not occur today (see also Castilla & Guiñez, 2000; DeVries, 1995). It is likely that the excursion of *Concholepas* outside South America was brief and temporary.

The third case is the turritellid gastropod genus *Incatella*, represented by the single living *I. cingulata* in the northern part of the Peruvian Province. The genus has a continuous fossil record from the Early Miocene onward in the Peruvian Province and a single probable occurrence (as *I. patagonica*) in the Monte Leon Formation (Early Miocene) of Argentina (DeVries, 2007a). If *I. patagonica* indeed belongs to this lineage, restriction of *Incatella* would have occurred after the Early Miocene.

The fourth case is the mussel *Semimytilus patagonicus*, formerly known as *S. algosus*. Today, this species is native only to the Peruvian Province, but the type specimen was collected in Argentina, where it no longer occurs (Signorelli & Pastorino, 2021). *S. patagonica* has also been recorded in temperate southern Africa, first in northern Namibia (Penrith & Kensley, 1970) but later spreading and becoming common on the west coast of South Africa (de Greef et al., 2013). It has generally been assumed that this small mussel was introduced to southern Africa by humans. The first African occurrence in relatively remote northern Namibia at 18° S latitude raises the possibility that, as with *Concholepas*, *Semimytilus* spread to Africa on its own. In any case, the refugial status of *Semimytilus* in the Peruvian Province rests on its single historical occurrence in Argentina.

## 6 GENERAL DISCUSSION

### 6.1 Unusual or unique features of the Peruvian Province

Compared with other climatically similar temperate biotas, the fauna and flora of the Peruvian Province exhibit a large number of highly unusual or even unique attributes. These are summarized in Table 1, together with the time of origin of these characteristics where known.

**TABLE 1:** Unusual features of the Peruvian Province Biota.

Feature	Time of origin
Absence of herbivores catching drift algae	Unknown origin
Absence of endothermic herbivores	Post‐Pliocene
Absence of large, cemented bivalves	Post‐Pliocene
Absence of large active cardiid bivalves	Post‐Pliocene
Absence of sedentary, deep‐burrowing, nonsiphonate bivalves	Post‐Pliocene
Absence of drilling naticid gastropods along most of coast	Post‐Pliocene
High incidence of dark‐colored shells	Pliocene
High abundance of herbivorous fishes	?Pliocene
High incidence of gastropods with a labral tooth	Early Miocene
High incidence of limpet‐like gastropods	Unknown origin
Reversed or humped latitudinal diversity gradient	Pleistocene
High magnitude of extinction	Middle Miocene, Pliocene

Of the twelve unusual conditions listed in Table 1, six are manifestations of a seventh, a high magnitude of extinction. Four of the extinction‐related conditions (absences of large cemented bivalves, active cardiids, deep‐burrowing siphonate bivalves, and drilling naticid gastropods) are associated with the sharp post‐Pliocene reduction in extensive areas of sheltered unconsolidated sediment and rocky areas. In the case of large cemented bivalves, no native or colonizing bivalve lineage functionally replaced large oysters, as did happen in California, where the huge cemented pectinid *Crassadoma gigantea* has a life habit convergent with that of giant Miocene oysters. No limpet‐like gastropod in the Peruvian Province has evolved the capacity to snag and consume drift algae, as large herbivores have done in California and South Africa. The prevalence of dark‐colored shells, perhaps linked to the presence of intertidal predatory sea stars, is a feature shared with California and with low‐shore gastropods in South Africa. The exceptionally high incidence of labral teeth among predatory muricid gastropods appears to be related to the high abundance of barnacles and mussels, which in turn is related to persistent high planktonic productivity. The high diversity of limpet‐like gastropods, many (but not all) of which cling tightly to hard substrates, might reflect the prevalence of wave‐exposed or current‐swept rocky habitats.

The previously unrecognized unique features of the benthic shallow water ecosystems of the Peruvian Province were not predicted. Instead, they came to light through an empiricle, unbiased examination of existing descriptive information on the sizes and composition of ecological guilds.

### 6.2 Guilds though time

As summarized in Table 2, trophic guilds in the Peruvian Province have undergone striking change due either to extinction or to the arrival of new members. Several guilds have disappeared completely since the Pliocene, including herbivorous marine mammals, cardiid suspension‐feeders (cockles), large isognomonids, large oysters, and coiled naticid gastropods (moon snails). Others witnessed a post‐Pliocene decline in diversity or maximum body size, including large patellogastropods, scallops, slow‐burrowing nonsiphonate bivalves, and durophagous marine mammals. Major post‐Miocene arrivals, with large effects on ecosystems, include kelps, large fissurellids (keyhole limpets), predatory sea stars, and perhaps large cancrid crabs. Guilds that have remained diverse, with large‐bodied living representatives, include barnacles, mussels, calyptraeid limpets, gastropods with a labral tooth, shallow‐burrowing siphonate bivalves, and predatory gastropods on soft bottoms.

**TABLE 2:** Peruvian Province guilds through time.

Guild	Miocene	Pliocene	Recent
*Primary producers*			
Large macroalgae	Unknown	Kelps	Kelps
Seagrasses	Present	Present	Highly localized
*Herbivores*			
Large patellogastropods	Present	Absent	Absent
Large fissurellids	Absent	Absent	Present
Large chitons	Unknown	Unknown	Present
Sea urchins	Unknown	Present	Present
Fishes	Unknown	Present	Present
Marine mammals	Present	Present	Present
Lizards	Unknown	Unknown	Present
*Suspension feeders*			
Large mussels	Present	Present	Present
Large barnacles	Present	Present	Present
Large calyptraeids	Present	Present	Present
Large tunicates	Unknown	Unknown	Present
Large isognomonids	Present	Present	Absent
Large oysters	Present	Present	Absent
Cardiid bivalves	Present	Present	Absent
Deep burrowing siphonate bivalves	Present	Present	Absent
Bioeroding bivalves	Present	Present	Present
Maximum size slow burrowing siphonate bivalves	97.5 mm	140 mm	115 mm
Maximum size large scallops	120 mm	138 mm	140 mm
Maximum size nonsiphonate burrowers	110 mm	73 mm	56 mm
*Benthic predators*			
Fishes	Present	pPresent	Present
Durophagous marine mammals	Present	Present	Present
Large crabs	Unknown	Unknown	Present
Mollusk‐eating sea stars	Unknown	Present?	Present
Drilling gastropods with labral tooth	Present	Present	Present
Coiled naticid gastropods	Present	Present	Absent
Maximum size large gastropods on soft bottoms	195 mm	100 mm	130 mm

Soft‐bottom and hard‐bottom ecosystems were affected in sharply contrasting ways. Six guilds that disappeared without replacement, and two additional guilds that suffered substantial decreases in diversity or maximum body size, are from soft bottoms or sheltered hardgrounds; another guild (durophagous mammals) likely existed on soft bottoms during the Miocene and Pliocene but today is represented by the southern sea otter, which forages primarily in the rocky intertidal zone. By contrast, all four guilds that were affected by the arrival of near colonists, and three of five guilds whose diversity and maximum body size were more or less stable, occur on hard bottoms, including the rocky intertidal zone. Hard‐bottom mussels and bioeroding bivalves should likely be added to this list, although firm data on fossil members of these guilds are not yet available. This contrast between hard and soft bottoms is highly statistically significant (*p* < .001 contingency test), was not previously recognized, and was discovered by exploration rather than by prior prediction and subsequent hypothesis testing.

### 6.3 Comparisons with other temperate biotas

The comparative histories of the world's temperate benthic marine biotas reveal striking and interesting contrasts. During the Miocene, the Southern Hemisphere biotas of Australasia and South America exchanged many lineages (Beu et al., 1997; Casadío et al., 2010) and produced many taxa that successfully colonized the North Pacific biota, which was then completely isolated from the North Atlantic. Although high levels of extinction affected all temperate Southern Hemisphere biotas, New Zealand and Argentina served as important biogeographic refuges for lineages that once also occurred in the Peruvian Province. Beginning in the Pliocene, the Peruvian Province began to receive important colonists from the north, including kelps, several herbivorous gastropods, and perhaps the sea star *Heliaster*. In the Northern Hemisphere, extinction reduced the diversity of species and eliminated some guilds in the North Atlantic, especially on the American side (Vermeij et al., 2008; Vermeij & Ruch, 2018), and the North Atlantic began to receive hundreds of colonists from the North Pacific by way of the newly opened Bering Strait and the Arctic Ocean (Vermeij et al., 2019). The role of donor biota thus shifted from the Southern Hemisphere to the North Pacific after the Miocene (Vermeij et al., 2019) as maximum sizes in most ecological guilds exceeded those on other temperate coasts (Vermeij, 2012b). Despite many topographic similarities between the west coasts of North and South America, the magnitude of extinction was low (less than 20%) in the North Pacific and very high in the Peruvian Province (61%–89% of molluscan species in the Plio‐Pleistocene).

In the number of innovations, the Peruvian Province is similar to southern Africa, Australasia, and the North Atlantic, each of which has four or five innovations, but it ranks far below the twenty‐four innovations identified in the temperate North Pacific (Vermeij, 2018). None of the seven South American innovations is unique to the Peruvian Province, in contrast to 15 of the 24 (63%) for the North Pacific. Two of the seven South American innovations (29%) spread to other regions: labral tooth in the Acanthina clade to the northeast Pacific, and limpet form and labral teeth in *Concholepas* briefly to South Africa. This percentage is comparable to the percentage of North Pacific innovations that spread to other regions (5 of 24, 23%), but several of the North Pacific novelties profoundly affected one or more areas, such as kelps, in all temperate regions and durophagy by walruses in the North Atlantic (Vermeij, 2018). These data reenforce the evolutionary and biogeographic dominance of the North Pacific relative to other coastal temperate marine ecosystems (Vermeij, 2018; Vermeij et al., 2019).

In short, the current status of the Peruvian Province as a highly productive, non‐refugial, recipient biota is the consequence of a unique historical trajectory characterized by a combination of severe extinction and changing contacts with other temperate regions. History matters; it affects species composition, species interactions, diversity, vulnerability to colonization, patterns of maximum body size, and biogeographic isolation.

### 6.4 Resilience

Although the biota of the Peruvian Province has been buffeted by repeated bouts of extinction and is today one of the most heavily exploited biotas by humans for food (Gutiérrez et al., 2016), it remains productive and functionally resilient. Most of its common species, whether they belong to lineages that have existed in the area for millions of years or are relative newcomers, evolved under conditions quite different from the ones under which they live today. Yet they are adaptable, and typically thrive under a wide range of circumstances. Perhaps owing to the generally high productivity of both the plankton and benthic algae, individuals grow fast and therefore quickly rebound from temporary losses. The Pliocene arrival of kelps from the North Pacific enriched the biota and likely made it more productive.

Kelps, in fact, exemplify this resilience well. These brown algae evolved in the North Pacific (Starko et al., 2019), with key adaptations that enable them to withstand intense herbivory by contemporaneously evolving desmostylian (and later by sirenian) mammals during the Early Oligocene (Kiel et al., 2024; Vermeij et al., 2019). In the Southern Hemisphere as well as in the North Atlantic, similarly adapted kelps became highly successful despite the absence of endothermic herbivores with large appetites.

## 7 CONCLUDING REMARKS

Biotas are regional collections of species whose diversity, interactions, and distribution are the consequence of evolution and geography, both of which are inherently historical. These species have properties; they are not merely names or numbers and therefore cannot be thought of as interchangeable automata. As products of history, biotas and the species and ecosystems in them have properties that cannot be understood simply by adducing present‐day explanatory factors. The biota of the Peruvian Province offers an exemplary system in which to investigate the all‐important role of history.

## AUTHOR CONTRIBUTIONS

**Geerat J. Vermeij:** Conceptualization (lead); writing – original draft (lead); writing – review and editing (lead). **Thomas J. DeVries:** Validation (equal); writing – review and editing (equal). **Miguel Griffin:** Validation (equal); writing – review and editing (equal). **Sven N. Nielsen:** Validation (equal); writing – review and editing (equal). **Diana Ochoa:** Validation (equal); writing – review and editing (equal). **Marcelo M. Rivadeneira:** Validation (equal); writing – review and editing (equal). **Rodolfo Salas‐Gismondi:** Validation (equal); writing – review and editing (equal). **Fernanda Valdovinos:** Funding acquisition (equal); writing – review and editing (equal).

## FUNDING INFORMATION

Work was funded by NSF grant number DEB‐2224915.

## CONFLICT OF INTEREST STATEMENT

None of the authors have competing interests.

## Data Availability

All the data are in the paper.
